# Social Factors Associated With CKM Stage—Is There Nothing New Under the Sun?

**DOI:** 10.1001/jamanetworkopen.2024.45251

**Published:** 2024-11-04

**Authors:** Amber E. Johnson, Jared W. Magnani

**Affiliations:** Section of Cardiology, Department of Medicine, University of Chicago Pritzker School of Medicine, Chicago, Illinois; Department of Medicine, University of Pittsburgh, Pittsburgh, Pennsylvania

This study by Zhu et al^1^ presents an analysis conducted using National Health and Nutrition Examination Survey data from 1999 to 2018 to identify the association between social determinants of health (SDOH) and cardiovascular-kidney-metabolic syndrome (CKM) by stage.^1^ The result is as one would expect: a gradient of CKM severity that increased as detrimental SDOH accumulated. For example, participants with 2 or more adverse SDOH were more likely to have advanced CKM stage than those with fewer than 2 adverse SDOH (age-standardized prevalence of advanced CKM, 15.8% [95% CI, 15.2%-16.5%] vs 10.5%[95% CI, 9.9%-11.1%]). Demonstrating these outcomes in a US population is important and validates similar findings in a Chinese cohort.^2^ Presentation of such findings is consistent with contemporary literature, particularly the epidemiological data shown in American Heart Association’s (AHA) presidential advisory focused on CKM.^3^

The findings of this analysis by Zhu et al^1^ are as expected based on abundant literature. We know from multiple analyses of population- and community-based cohorts that social risk factors have associations with health outcomes. Associations between SDOH and heterogeneous clinical constructs, such as CKM, are likely complicated. Social risk factors are the SDOH that adversely impact health outcomes and can include low level of educational attainment, poverty, and food insecurity,^4^ as demonstrated in the study by Zhu et al.^1^ To their credit, Zhu et al^1^ completed a decision tree analysis that revealed low employment status (advanced CKM prevalence, 18.8% [95% CI, 17.7%-20.1%] among unemployed participants vs 11.4% [95% CI, 11.0%-11.9%] among employed participants), inadequate health insurance (advanced CKM prevalence, 16.5% [95% CI, 15.7%-17.4%] among participants with government or no insurance vs 11.0% [95% CI, 10.5%-11.6%] among those with commercial insurance), and low family income-to-poverty ratio (advanced CKM prevalence, 16.1% [95% CI, 15.4%-16.8%] among participants with income <300% the poverty level vs 10.1% [95% CI, 9.5%-10.7%] among those with income ≥300% the poverty level) as the key social risks associated with CKM stage.

The association between social risk factors and CKM should be reviewed in the context of increasing support for CKM as a relevant and pressing clinical outcome. Indeed, a complementary analysis used the same National Health and Nutrition Examination Survey data to stratify probability of CKM by social risk profile.^5^ Current and future trends in the field will likely include measuring CKM; however, translating such risk into actionable clinical strategies is yet to be seen. The AHA Predicting Risk of Cardiovascular Disease (CVD) Events (PREVENT) models reflect the interrelatedness and upstream associations of CKM conditions with CVD risk. PREVENT provides a critical next step forward to prioritize primary prevention across the spectrum of CKM and equitably improve health in the population. To capture neighborhood-level risk, PREVENT uses an optional zip code add-in to further stratify patients by risk of heart disease and CKM.^6^ Emphasis on screening for CKM will likely continue to increase as clinicians and public health organizations focus more on CKM as a clinical entity.

As the field of health equity continues to evolve, researchers looking to advance the science will need to incorporate analyses that not only implicate disparities but also show meaningful avenues for change. The analysis presented by Zhu et al^1^ attempted to understand hierarchical associations of SDOH with CKM risk. Using decision tree analysis, they suggest that addressing employment and income status could serve as potential levers of change. Sex was also notably associated with CKM. Future approaches may also consider principal components or hierarchical regression analyses.

How might a patient or clinician address female sex, low education level, or low family income as a barrier to care? The analysis by Zhu et al^1^ begins to show patterns of risk enhancement but is lacking in tangible solutions to improve outcomes. CKM is a conglomerate diagnosis spanning multiple organ systems. Thus, on the one hand, solutions could be multiplicative in their impact. On the other hand, researchers will need to use caution to disaggregate associations and make useful recommendations to determine both statistical and clinical significance.

Zhu and colleagues^1^ conclude that “addressing these adverse factors might be crucial for the prevention and treatment of CKM syndrome.” Others have found that screening for social risk factors can successfully lead to the provision of relevant assistance.^7^ Screening for unemployment, low family income, food insecurity, or housing status will next require innovative solutions for patient-and community-facing strategies to reduce the risk of CKM and adverse outcomes.
